# The work pattern of personal care workers in two Australian nursing homes: a time-motion study

**DOI:** 10.1186/1472-6963-12-305

**Published:** 2012-09-06

**Authors:** Si-Yu Qian, Ping Yu, Zhen-Yu Zhang, David M Hailey, Pamela J Davy, Mark I Nelson

**Affiliations:** 1 School of Information Systems and Technology, University of Wollongong, New South Wales, 2522, Australia; 2 School of Mathematics and Applied Statistics, University of Wollongong, New South Wales, 2522, Australia

## Abstract

**Background:**

The aim of the study is to describe the work pattern of personal care workers (PCWs) in nursing homes. This knowledge is important for staff performance appraisal, task allocation and scheduling. It will also support funding allocation based on activities.

**Methods:**

A time-motion study was conducted in 2010 at two Australian nursing homes. The observation at Site 1 was between the hours of 7:00 and 14:00 or 15:00 for 14 days. One PCW was observed on each day. The observation at Site 2 was from 10:00 to 17:00 for 16 days. One PCW working on a morning shift and another one working on an afternoon shift were observed on each day. Fifty-eight work activities done by PCWs were grouped into eight categories. Activity time, frequency, duration and the switch between two consecutive activities were used as measurements to describe the work pattern.

**Results:**

Personal care workers spent about 70.0% of their time on four types of activities consistently at both sites: direct care (30.7%), indirect care (17.6%), infection control (6.4%) and staff break (15.2%). Oral communication was the most frequently observed activity. It could occur independently or concurrently with other activities. At Site 2, PCWs spent significantly more time than their counterparts at Site 1 on oral communication (Site 1: 47.3% vs. Site 2: 63.5%, P = 0.003), transit (Site 1: 3.4% vs. Site 2: 5.5%, P < 0.001) and others (Site 1: 0.5% vs. Site 2: 1.8%, P < 0.001). They spent less time on documentation (Site 1: 4.1% vs. Site 2: 2.3%, P < 0.001). More than two-thirds of the observed activities had a very short duration (1 minute or less). Personal care workers frequently switched within or between oral communication, direct and indirect care activities.

**Conclusions:**

At both nursing homes, direct care, indirect care, infection control and staff break occupied the major part of a PCW’s work, however oral communication was the most time consuming activity. Personal care workers frequently switched between activities, suggesting that looking after the elderly in nursing homes is a busy and demanding job.

## Background

The growing ageing population has resulted in an increasing number of residents, especially the very old and frail, in residential aged care facilities (RACFs) [1]. This requires an increase in the number and intensity of the aged care services. The situation is worsened by a chronic shortage of direct care workers, on whom these people rely to live [2]. All of this represents a big challenge to the delivery of aged care services in RACFs.

An important strategy to address this challenge is to effectively design work activities to optimally deliver aged care services. This requires a basic knowledge about which work activities are currently undertaken by direct care workers and how much time each activity actually takes to meet a resident’s care needs.

Personal care workers (PCWs) make up the largest proportion (70%) of the direct care workers in RACFs. Because of the challenge of attracting registered nurses (RNs), the number of PCWs is increasing steadily in Australia [3]. Personal care workers have a minimum qualification of Certificate III in Aged Care awarded by the Technical and Further Education (TAFE) college system in Australia. They are the major providers of personal care to residents, especially the activities of daily living (ADL) which are one of the important care needs supported by the Aged Care Funding Instrument (ACFI) [4]. The ACFI assesses the day-to-day core care needs of a resident to determine the level of subsidy.

Work sampling and time-motion techniques have been applied extensively to measure the work pattern in healthcare settings [5-12]. The former has a relatively small cost, but is not able to capture some important information such as the duration of an activity because the observation is not continuous [13]. The latter allows precise time to be recorded for each activity, but this is labor-intensive and costly [14].

Previous studies have investigated the work pattern in hospital settings [6-8,10,11]. For example, Cornell *et al.*[7] inspected nurses’ workflow and their computer use in two acute care medical-surgical units in a general hospital in the USA. They found that nurses frequently switched between activities and the duration of most of the activities was very short and highly variable. A recent study described the work activities of bedside intensive care unit nurses in a private hospital in Australia [11]. Nurses spent most of their time on direct care and indirect care and they undertook two activities simultaneously for almost half of their time.

Although there has been much research on the work pattern in hospital settings, few such studies have been undertaken in RACFs. Among the studies undertaken in RACFs, some only focused on particular activities (e.g. bathing-related care) [5,12,15]. Munyisia *et al.*[9] examined the time expenditure on different types of activities performed by direct care workers by conducting a work sampling study in a high-care house and a low-care house of an RACF. They found that in both houses, oral communication was the most time-consuming activity (32.4%-51.9%). This study provides a comprehensive overview of what the direct care workers do and how they spend their working time, however it was confined to a single RACF and because it is a work sampling study, the duration of each activity or the switches which occur between activities could not be determined.

This study aims to accurately describe the work pattern of PCWs in two high-care RACFs. These are similar to nursing homes in the USA in terms of the level of care provided to residents. Previous studies used different measurements to describe the work pattern [7,10,11,16,17]. The commonly used measurements are activity time, frequency, duration and the switch between two consecutive activities.

Activity time is expressed by two parameters: (1) the time an activity takes over an eight-hour shift and (2) the percentage of time used to complete an activity in relation to the total amount of time for all activities. Activity frequency is the number of occurrences of an activity during a set period of time (e.g. an hour). Activity duration, usually assessed in seconds, is the length of time continuously spent on an activity. It is presented as a mean with standard deviation to indicate its variability. The switch between two consecutive activities includes the number of occurrence of a switch and the direction of this switch. These four measurements were used in this study to describe a PCW’s work pattern.

## Methods

### Settings

A time-motion observational study was conducted at two nursing homes. The first nursing home was located in Sydney and was owned by a not-for-profit organization which operates 23 RACFs. The observation was conducted in a 32-bed high-care wing (Site 1) staffed by one half-time and four full-time PCWs and one RN. The other nursing home was a stand-alone, not-for-profit facility in Newcastle with 108 beds. The observation was conducted in a 25-bed high-care wing (Site 2) in which three PCWs and one RN took care of 23 residents (two beds were empty at the time of the study).

### Classification of personal care workers’ activities

The observational study requires a predefined classification of activities. Our research team has developed and applied an activity classification system of direct care workers in a longitudinal work sampling study conducted in an Australian nursing home [9,18-20]. This work activity classification system was further developed and revised through three focus group discussions with three researchers (including the researchers who developed it) and three RNs who had extensive experience working in aged care.

The final classification system contains 58 activities grouped into eight categories: direct care, indirect care, infection control, documentation, transit, staff break, oral communication and other activities not included in the previous categories. The activities in each category are presented in Table 1.

**Table 1:** Classification of personal care workers’ activities

**Category**	**Activities**
Direct care	Physical Assessment.
	Routine hygiene (e.g. daily shower or wash).
	Continence related hygiene (e.g. shower or wash following pad change).
	Oral Care.
	Shave or grooming.
	Toileting - prompted by a resident.
	Toileting - prompted by a personal care worker.
	Pad check.
	Pad change.
	Scheduled toileting.
	Dressing a resident.
	Resident mobility; passive & active exercises; turning a resident in bed.
	Medication administration.
	Specimen collection; urine collection.
	Assisting a resident with eating and drinking (include feeding systems).
	Assisting a resident with food (e.g. cutting up food, uncovering food or delivery of food).
	Care of the deceased; laying out.
	Assisting a resident with hand washing following the use of toilet.
	Assisting a resident with transfer to and from a bed, a chair, etc.
	Transferring a resident to or from dining room or board room.
	Weighing a resident.
	Assisting a resident to receive a phone call.
	Attending to a resident call for assistance.
Indirect care	Equipment set up (e.g. sling set up, shower chair set up).
	Resident shower set up (e.g. preparing shampoo, towel or body lotion).
	Bed making routine.
	Changing a bed following an incontinent episode.
	Cleaning up spills following an incontinent episode.
	Re-stocking supplies to a trolley.
	Re-stocking supplies to a resident's cupboard.
	Transporting linen to and from laundry.
	Transporting clinical waste for disposal.
	Using or cleaning up bed pans.
	Emptying a resident's meal plate.
	Collecting pads from a storage cupboard.
	Collecting a resident's clothes from his or her cupboard; putting clothes back to the cupboard.
	Sorting and putting a resident's clothes to his or her room.
Infection control	Putting on personal protective equipment.
	Taking off personal protective equipment.
	Alcohol hand washing (related to toileting or pad change).
	Alcohol hand washing (unrelated to toileting or pad change).
	Water hand washing (related to toileting or pad change).
	Water hand washing (unrelated to toileting or pad change).
Documentation	Locating or collecting a resident's records.
	Taking a photo of a resident.
	Reviewing or writing resident's clinical information; reading notes; viewing results.
	Putting records back to filing area.
Transit	Standing or walking in the corridor between activities.
Staff break	Personal errands (off unit chores; meal break; making personal telephone call).
Oral communication	Asking for assistance from another personal care worker.
	Assisting another personal care worker to do his or her work.
	Participating in-service training.
	Communication of information about a resident (external).
	Communication of information about a resident (internal).
	Communicating with a resident.
	Communicating with a resident's family.
	Receiving a phone call; making a phone call.
Others	Other tasks not included.

### Ethical approval

Ethical approval was granted by the Human Research Ethics Committee of the University of Wollongong based on written approval given by the two participant aged care organizations which run the two nursing homes.

### Inter-rater reliability

Our observation was conducted by a single observer. To ensure the reliability of the observation process, our observer and a second observer, who has extensive experience in conducting observational studies, independently observed and recorded the same activities of four PCWs for a period of four hours. Then a comparison of two hours of their records was conducted and discussed. A minimum agreement of more than 95% was achieved in the two records, suggesting the inter-rater reliability is adequate according to Pelletier and Duffield [21].

### Data collection

The observation was performed in 2010. Before the observation, the nursing manager at each site introduced the observer to the RNs and the PCWs. On each observational day, the observer arrived at the site 15 minutes before the start of the observation to identify one of the PCWs for observation, using convenience sampling. The observer tried to observe different PCWs on different days to maximize the number of participants. At the start, the observer explained the purpose and procedure of the observation to the PCW. Only after written consent was given by the participant, was the observation conducted. A clinical handheld was used to record the observational data on an Excel spreadsheet.

For the first seven days of data collection at Site 1 only the start time was recorded for the observed activities. Because the start time of the current activity is the end time of the previous activity, it was not necessary to record the end time. The observer noticed that a PCW might only be speaking or might be performing some other activity concurrently. In order to correctly record oral communication time, the data collection protocol was modified to include both the start time and end time of an oral communication activity, and the concurrently performed activity. At Site 2, both start and end times of oral communication activities were recorded from the beginning.

At Site 1, a total of 11 PCWs were observed over a period of 14 days (three of the PCWs were observed twice). The observation was between the hours of 7:00 and 14:00 or 15:00 on each day, depending on the observed PCW’s finishing time.

At Site 2, a total of 27 PCWs were observed over 16 days (five were observed twice). The observation was from 10:00 to 17:00. On each day, a morning shift PCW was observed first. After this individual finished work at 14:00 or 15:00, an afternoon shift PCW was observed.

### Data analysis

The data were analyzed in Microsoft Excel 2007, SPSS version 18.0 (SPSS inc., Chicago, IL, USA) and R version 2.12.1 [22]. The duration of each activity was calculated in Excel. Analysis concerning oral communication at Site 1 was based on the last seven days of observation since the end time was not recorded during the first seven observational days. A Z test was used to compare the percentage of time spent on each category of activities between the two sites. A Pearson’s chi square test was used to determine the difference between the two sites in the number of activities which fell into different duration groups. Statistical significance was assumed when P < 0.05.

## Results

Fifty-one of the designated 58 activities were observed at Site 1. The seven activities which were not observed are: ‘care of the deceased or laying out’, ‘assisting a resident to receive a phone call’, ‘re-stocking supplies to a resident’s cupboard’, ‘using or cleaning up bed pans’, ‘sorting and putting a resident’s clothes to his or her room’, ‘participating in-service training’ and ‘taking a photo of a resident’. Fifty-five of the designated activities were observed at Site 2. The three activities which were not observed are: ‘cleaning up spills following an incontinent episode’, ‘re-stocking supplies to a resident’s cupboard’, and ‘taking a photo of a resident’.

A total of 173 hours of observation and 11,283 events were recorded. Table 2 shows the time spent on each category of activities, combining data from the two sites. The percentages do not sum to 100% because oral communication may occur either by itself or simultaneously with an activity from one of the other seven categories. This means that the percentage of time spent on oral communication has overlaps with the other categories.

**Table 2:** Time spent on each category of activities, combining the two sites

**Categories**	**Time (%)**	**95% Confidence intervals**	
Direct care	30.7	28.7	32.8
Indirect care	17.6	16.3	18.8
Infection control	6.4	5.8	7.1
Documentation	3.1	2.5	3.7
Transit	4.6	3.9	5.2
Staff break	15.2	11.8	18.6
Oral communication	59.2	53.7	64.6
Others	1.2	0.8	1.6

### Activity time

At Site 1, 81 hours of observation were recorded and at Site 2, 92 hours were recorded. Table 3 presents the time, frequency and duration by activity category at each site.

**Table 3:** Time, frequency and duration by activity category at Site 1 and Site 2

**Category**	**Site**	**Time**				**Frequency per hour**	**Duration (seconds)**			
		**%**	**95% Confidence intervals**		**8-hour shift (h:m:s)**		**Mean**	**Standard deviation**	**95% Confidence intervals**	
Direct care	1	30.9	28.7	33.0	2:28:05	14.5	75.6	143.5	67.4	83.8
	2	30.7	28.1	33.2	2:27:07	19.6	56.5	56.7	53.8	59.1
Indirect care	1	16.7	15.5	17.9	1:19:55	10.6	56.1	54.5	52.5	59.8
	2	18.4	16.7	20.2	1:28:22	13.2	50.5	46.8	47.9	53.2
Infection control	1	5.9	5.2	6.6	0:28:25	5.3	40.2	61.5	34.3	46.1
	2	6.8	6.1	7.6	0:32:46	8.8	28.2	24.5	26.5	29.9
Documentation	1	4.1 ^a^	3.4	4.8	0:19:31	3.2	45.5	82.4	35.4	55.6
	2	2.3 ^b^	1.7	3.0	0:11:13	1.4	58.8	80.7	44.9	72.7
Transit	1	3.4 ^a^	3.0	3.9	0:16:29	3.0	41.3	46.7	35.4	47.2
	2	5.5 ^b^	4.5	6.6	0:26:34	3.0	67.1	84.4	57.0	77.1
Staff break	1	14.6	10.9	18.4	1:10:17	0.6	880.1	817.5	642.7	1117.5
	2	15.7	11.0	20.4	1:15:24	0.7	815.0	845.4	603.8	1026.2
Oral communication	1	47.3 ^a^	39.3	55.3	3:47:00	18.8	90.7	140.9	80.0	101.5
	2	63.5 ^b^	56.6	70.4	5:04:40	25.8	88.9	173.4	81.9	95.8
Others	1	0.5 ^a^	0.3	0.7	0:02:24	0.2	96.3	70.0	57.6	135.1
	2	1.8 ^b^	1.1	2.5	0:08:46	0.6	110.3	118.5	78.3	142.3

^a,b^Indicate significant difference between the two sites in the percentage of time spent on this category of activities (P < 0.05).

At Site 1, the most time-consuming direct care activity was ‘assisting a resident with eating and drinking (include feeding systems)’ at 35 minutes over an eight-hour shift. At Site 2, however, ‘assisting a resident with transfer to and from a bed, a chair, etc.’ was the most time-consuming direct care activity, taking 29 minutes over an eight-hour shift. At both sites, ‘equipment set up (e.g. sling set up, shower chair set up)’ took the most indirect care time (Site 1: 31 minutes, Site 2: 38 minutes). Most of the oral communication time was spent on ‘communication of information about a resident (internal)’ (Site 1: 1 hour and 53 minutes, Site 2: 2 hours and 42 minutes) and ‘communicating with a resident’ (Site 1: 1 hour and 51 minutes, Site 2: 2 hours and 4 minutes).

No statistically significant difference between the two sites was found in the time spent on direct care, indirect care, infection control or staff break, and these activities took approximately 70.0% of the working time (Site 1: 68.1%, Site 2: 71.6%). As shown in Table 3, PCWs at Site 2 spent significantly more time than their counterparts at Site 1 on oral communication (Site 1: 47.3% vs. Site 2: 63.5%, P = 0.003), transit (Site 1: 3.4% vs. Site 2: 5.5%, P < 0.001) and others (Site 1: 0.5% vs. Site 2: 1.8%, P < 0.001). They spent less time, however, on documentation (Site 1: 4.1% vs. Site 2: 2.3%, P < 0.001).

### Activity frequency

In one hour, 56 events occurred at Site 1 and 73 events occurred at Site 2. The most frequently occurring activity was oral communication, followed by direct care and indirect care (Table 3).

### Activity duration

**Figure 1:**
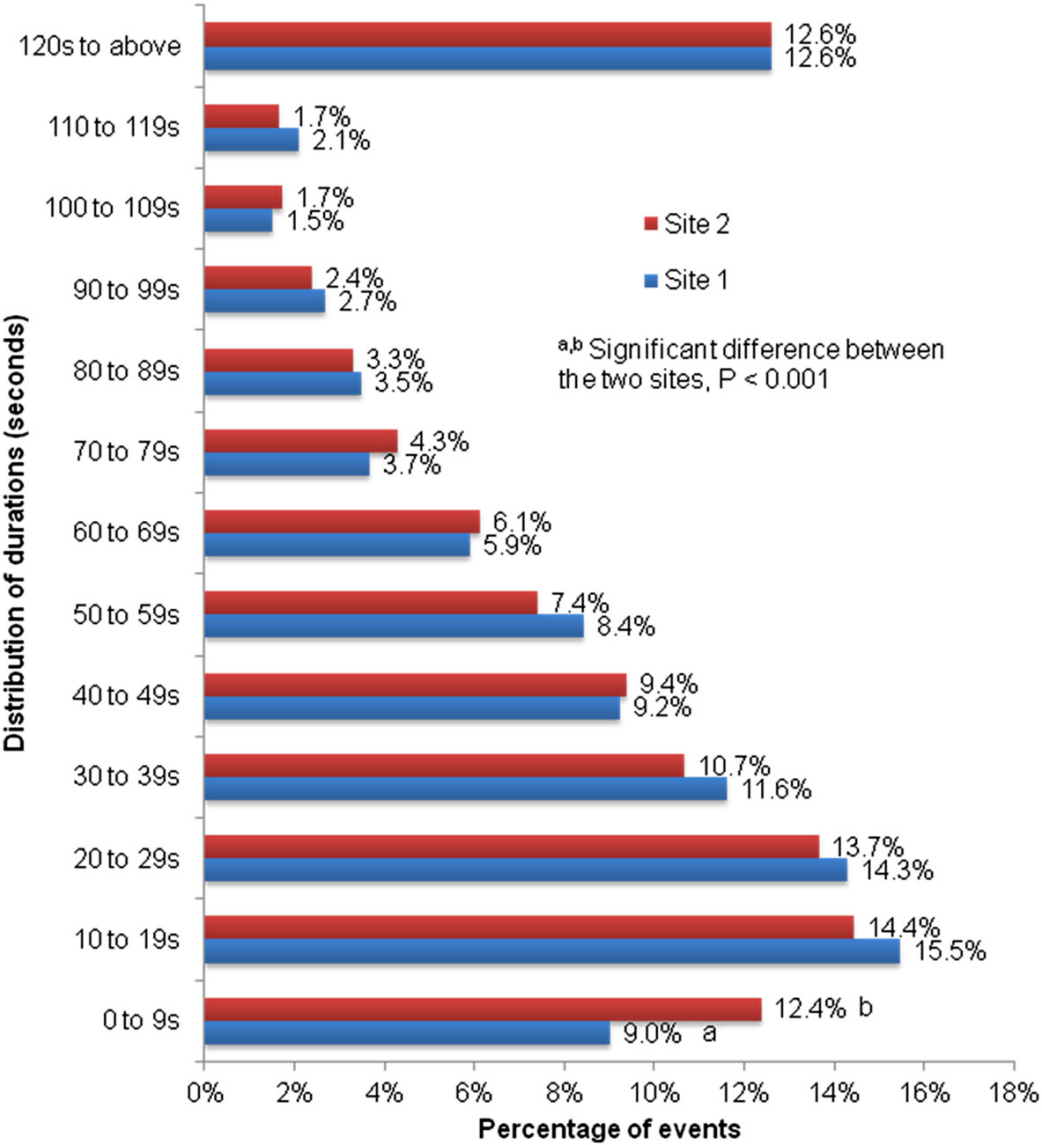
Distribution of duration at Site 1 and Site 2.

From the mean and standard deviation shown in Table 3, the activity duration was very short and varied dramatically. The short activity duration is also shown in Figure 1. Of the 3,679 events recorded at Site 1 (excluding the 889 oral communication events which occurred in the first seven days), 9.0% were completed in less than 10 seconds, which was significantly less than at Site 2 (12.4% of 6,715 events, P < 0.001). 15.5% of the events recorded at Site 1 and 14.5% at Site 2 took between 10 and 19 seconds. Overall, more than two-thirds of the observed events at both sites had a duration of less than 1 minute.

### Switch between two consecutive activities

A PCW frequently switched from one activity to another. On average, 49.8 switches between two consecutive activities were observed in an hour at Site 1 and 62.5 switches were observed at Site 2. A PCW switched from one activity to another at a rate of one per minute. Most of the switches were within or between oral communication activities, direct care activities and indirect care activities. The directions of the most frequently observed switches were similar, as were the number of these switches at both sites (Table 4).

**Table 4:** Direction and number of the most frequently observed switches between two consecutive activities

**First activity**	**Second activity**	**Number of switches per hour**
Communicating with a resident.	Communication of information about a resident (internal).	1.6
Communication of information about a resident (internal).	Communicating with a resident.	1.6
Equipment set up (e.g. sling set up, shower chair set up).	Assisting a resident with transfer to and from a bed, a chair, etc.	1.1
Communicating with a resident.	Communicating with a resident.	0.9
Taking off personal protective equipment.	Water hand washing (related to toileting or pad change).	0.8
Communicating with a resident.	Equipment set up (e.g. sling set up, shower chair set up).	0.6
Assisting a resident with transfer to and from a bed, a chair, etc.	Equipment set up (e.g. sling set up, shower chair set up).	0.9

## Discussion

In this study 56 out of the 58 designated activities performed by PCWs at two Australian nursing homes were measured. The activities were classified into eight categories: direct care, indirect care, infection control, documentation, transit, staff break, oral communication and others. In comparison with a previous study in a single nursing home [9], this study provides a much more accurate and complete picture of how PCWs spend their time on work activities described in the following terms: actual time taken over an eight-hour shift, the time spent on it as a percentage of the time consumed by all of the observed activities, the activity frequency and duration. It also provides data on the switches between two consecutive activities. Our findings will be useful for nursing managers to understand how PCWs work and what the workload actually is in looking after residents with high-care needs in nursing homes. Although the care needs of the residents represented an uncontrolled variable in what was a natural setting, it appeared that a PCW’s workload looking after residents and meeting their care needs was high in both nursing homes.

Personal care workers spent 30.7% of their time on direct care. This is less than the finding (40.2%) from a previous study by Munyisia *et al.*[9] which was also conducted in an Australian nursing home. Indirect care consumed 17.6% of PCWs’ time, which is almost twice as the time (8.9%) obtained in the study by Munyisia. The difference in time may be caused by the different study design. For example, our study used time-motion technique to collect data while their study used a work sampling technique. The other possibility is that the differences are due to differences in care systems and practices in different nursing homes. Furthermore, in this study the percentage of time was calculated from the actual duration of activities, whereas their results were based on the frequency of occurrence of activities.

Further analysis needs to be conducted to understand how indirect care activities support direct care. It is also necessary to examine how direct care activities distributed throughout a shift and whether the direct care activities were spread out evenly over an hour or performed in quick succession, for example, at the beginning of the hour. This can make a significant difference to residential care, as was also mentioned in a previous study [10].

Communication with a resident and communication of information about a resident are the prime oral communication activities. This may be an indication that the PCWs had made an effort to spend time interacting with residents (e.g. explaining the care to a resident in order to receive cooperation from the resident) and cooperate with the working partners to provide care. The content of the oral communication and the way it is conducted may be among the critical elements which most affect the quality of care.

Personal care workers not only spent a great deal of time on oral communication, but also frequently switched between oral communication, direct care and indirect care activities. This may indicate that oral communication is one of the important activities which support direct care and indirect care.

Although the observational time periods at the two nursing homes were different (Site 1: 7:00 to 14:00 or 15:00, Site 2: 10:00 to 17:00), no statistically significant difference was found in the time spent on direct care, indirect care, infection control or staff break. These activities account for about 70.0% of a PCW’s working time. This suggests that apart from the unavoidable breaks which all staff must take, these activities represent the core of PCWs' workload. Nursing managers need to consider this finding carefully when allocating tasks, staff number and skill mix on a shift.

Personal care workers at Site 1 spent significantly less time on oral communication than their counterparts at Site 2. This may be associated with the age and ethnicity of the PCWs. Most of those at Site 1 were 20 to 30 years old and from a non-English speaking background, whereas PCWs at Site 2 were local and aged between 35 and 55. As the PCWs at Site 2 had the same language and cultural background as the residents, oral communication was less of a challenge than it was for the PCWs at Site 1.

The often short duration of activities and the quick and frequent switching between activities caused extreme busyness and some stress. The practical routine and familiarity with the residents and their individual needs help the PCWs arrange their work to cope with this. This routine and familiarity with the residents can facilitate the work. This was also found in a previous study [23].

Although routine and familiarity may support their work, a PCW does have to think about what to do next while performing the task at hand. Working in such a busy environment may lead to a cognitive overload, which may cause job fatigue and contributing, in turn, to nursing burn out. Therefore, nursing managers may need to consider which level of workload is appropriate for a PCW working in a nursing home.

Among the designated 58 work activities of PCWs, 56 were observed, suggesting that our activity classification system reflects a PCW’s work activities in Australian nursing homes and provides a good reference for other studies of work activities in nursing homes.

### Limitations

The benefit of using a single observer is the potential consistency of the observations [24], however it may also cause systematic errors in observation. We addressed this potential limitation through an inter-rater reliability comparison study, which provided satisfactory results. There may be a ‘Hawthorn effect’ [13] (the participants might change their work behavior under the observation) from PCWs being observed continuously, however we found that in the busy nursing home working environment, PCWs had to focus on their job and very soon ignored the existence of the observer. This was also found in previous studies [25,26].

## Conclusions

We described the work pattern of PCWs in two Australian nursing homes. The work activities were examined using the following measurements: activity time, frequency, duration, and the switch between two consecutive activities. Fifty-six out of 58 designated work activities grouped into eight categories were observed. We found that direct care, indirect care, infection control and staff break were the major part (70.0%) of the work and there was no statistically significant difference between the two nursing homes in the time spent on these activities. More than two-thirds of the observed activities at both sites had a very short duration-- less than 1 minute. Personal care workers frequently switched within or between oral communication, direct care and indirect care activities.

Our findings are useful for nursing managers for staff performance appraisal, task allocation, scheduling and cost estimation. The information may also help to design effective aged care services and provide possible research directions in nursing homes. Furthermore, it provides evidence for the government in funding allocation by accurately measuring the amount of time needed in conducting each category of care activities to meet a resident’s relevant care needs. Further research on how indirect care activities support direct care and how oral communication supports other types of care are needed.

## Abbreviations

RACFs: Residential aged care facilities; PCWs: Personal care workers; RNs: Registered nurses; TAFE: Technical and Further Education; ADL: Activities of daily living; ACFI: Aged Care Funding Instrument.

## Competing interests

The authors declare that they have no competing interests.

## Authors' contributions

PY was responsible for the study conception and design. ZYZ performed data collection. SYQ, PD and MN were responsible for data analysis. SYQ drafted the manuscript. PY, DH, PD and MN made critical revisions to the paper for important intellectual content. All authors read and approved the final manuscript.

## Pre-publication history

The pre-publication history for this paper can be accessed here:

http://www.biomedcentral.com/1472-6963/12/305/prepub
